# One week pre-operative oral antibiotics for percutaneous nephrolithotomy reduce risk of infection: a systematic review and meta-analysis

**DOI:** 10.1590/S1677-5538.IBJU.2022.0544

**Published:** 2022-12-15

**Authors:** Alexandre Danilovic, Thalita Bento Talizin, Fabio Cesar Miranda Torricelli, Giovanni S. Marchini, Carlos Batagello, Fabio C. Vicentini, Willaim C. Nahas, Eduardo Mazzucchi

**Affiliations:** 1 Departamento de Urologia Universidade de São Paulo Hospital das Clínicas São Paulo SP Brasil Departamento de Urologia, Universidade de São Paulo Hospital das Clínicas - HCUSP, São Paulo, SP, Brasil;; 2 Departamento de Urologia Hospital Alemão Oswaldo Cruz São Paulo SP Brasil Departamento de Urologia, Hospital Alemão Oswaldo Cruz, São Paulo, SP, Brasil;; 3 Departamento de Urologia Faculdade de Medicina Universidade de São Paulo São Paulo SP Brasil Departamento de Urologia Faculdade de Medicina da Universidade de São Paulo - FMUSP, São Paulo, SP, Brasil

**Keywords:** Anti-Bacterial Agents, Drug Therapy, Kidney Calculi, Nephrolithotomy, Percutaneous

## Abstract

**Purpose:**

The aim of this meta-analysis is to assess the efficacy of extended dose of preoperative antibiotics to reduce infectious risk in patients undergoing percutaneous nephrolithotomy (PCNL).

**Materials and Methods:**

A literature search for prospective case-control studies or randomized controlled trials was done. PICO framework was used. Population: adult patients that underwent to PCNL; Intervention: extended dose preoperative antibiotic prophylaxis before PCNL; Control: short dose preoperative antibiotic prophylaxis before PCNL; and Outcome: systemic inflammatory response syndrome (SIRS) or sepsis, fever after PCNL and positive intraoperative urine and stone culture. This meta-analysis was registered in PROSPERO database under the number: CRD42022359589.

**Results:**

Three RCT and two prospective studies (475 patients) were included. SIRS/sepsis outcome was retrieved from all studies included. Seven days preoperative oral antibiotics for PCNL was a protective factor for developing SIRS/sepsis (OR 0.366, 95% CI 0.234 - 0.527, p < 0.001). There was no statistical association between seven-day use of antibiotics and fever (OR 0.592, 95% CI 0.147 – 2.388, p = 0.462). Patients who received seven days preoperative antibiotics had lower positive intraoperative urine culture (OR 0.284, 95% CI 0.120 – 0.674, p = 0.004) and stone culture (OR 0.351, 95% CI 0.185 – 0.663, p = 0.001) than the control group.

**Conclusion:**

one week of prophylactic oral antibiotics based on local bacterial sensitivity pattern plus a dose of intravenous antibiotics at the time of surgery in patients undergoing PCNL reduces the risk of infection.

## INTRODUCTION

Percutaneous nephrolithotomy (PCNL) is the current gold standard treatment for kidney stones > 20 mm ( 1 ). Although effective, PCNL is associated with complications such as prolonged urinary leakage in up to 10% and blood transfusion in up to 7% of the patients ( 2 - 5 ). Approximately 10% of the patients develop a postoperative fever after PCNL, while sepsis is reported in 0.3% to 0.5% ( 5 , 6 ). Despite being rare, urosepsis is a life-threatening complication of PCNL, and every effort should be made to prevent its occurrence.

There is no specific recommendation for a preoperative antibiotic regimen in patients undergoing PCNL due to insufficient data ( 1 , 7 ). Previously published meta-analyses evidenced significant heterogeneity between included studies. Retrospective and prospective studies were analyzed together, preoperative, and postoperative antibiotic regimens were compared in the same meta-analysis, and duplicates were included making it impossible to determine the role of preoperative antibiotics( 8 - 10 ). There is no consensus on the definition of high infectious risk patients. Several possible risk factors for infection were investigated. Patient positioning in PCNL, tract size, obesity and solitary kidney do not seem to impact infectious rates ( 11 - 14 ). Some investigators consider high risk for infection stone size ≥ 20 mm and/or dilation of the collecting system with sterile urine. However, other authors define high infectious risk for PCNL as those with a positive preoperative urine culture within three months of the planned procedure or an indwelling stent or nephrostomy tube at the time of surgery, without considering stone size or dilation of the collecting system ( 15 - 17 ). As the definition of high infection risk is unclear, this study aims to perform a high-quality meta-analysis using only prospective studies to define the role of preoperative antibiotics in patients undergoing PCNL.

## MATERIALS AND METHODS

### Identification and Eligibility of Trials

The meta-analysis protocol was registered on the PROSPERO database on September 22, 2022 (CRD42022359589). This review was conducted according to PRISMA (preferred reporting items for systematic reviews and meta-analyses) statement ( 18 ). We selected prospective studies and randomized controlled trials (RCT) that compared extended to short-dose preoperative antibiotic prophylaxis in patients undergoing PCNL. On May 2022, the key words “percutaneous nephrolithotomy” and “antibiotic” were searched on EMBASE, PubMed, and Web of Science platforms. Retrospective studies, case reports, case-control studies, letters to the editor, editorials, congress abstracts, and studies in patients < 18 years old were excluded.

### Development of Prospective Meta-analysis Protocol

The PICO (population, intervention, control, and outcome) framework was agreed upon before the collection of data:

- Population: adult patients that underwent PCNL;
- Intervention: extended dose preoperative antibiotic prophylaxis before PCNL;
- Control: short dose preoperative antibiotic prophylaxis before PCNL; and
- Outcome: systemic inflammatory response syndrome (SIRS) or sepsis, fever after PCNL, positive intraoperative urine culture, and stone culture.

### Outcomes and Comparisons

The primary outcome measure was SIRS or sepsis after PCNL. Primary comparison investigated extended dose preoperative antibiotic prophylaxis vs. short dose preoperative antibiotic prophylaxis before PCNL. Secondary outcome measures investigated included fever after PCNL, positive intraoperative urine, and stone cultures. We considered extended dose the use of preoperative antibiotics for seven days before PCNL and short dose for ≤ 2 days. SIRS or sepsis were defined according to each study ( 19 , 20 ).

### Assessment of risk of bias in included studies

Risk of bias assessments were done independently by two of the investigators with agreement, without discrepancy. The risk of bias for each RCT was assessed using version 2 of the Cochrane Risk of Bias Assessment Tool (RoB 2). RoB 2 is structured into domains of bias (trial design, conduct, and reporting results) and classified as unclear, low, and high risk ( 21 ). The risk of bias for each prospective study was defined using The Risk of Bias In Non-randomized Studies of Interventions (ROBINS-I), recommended by the Cochrane Scientific Committee. ROBINS-I is structured into the selection of patients, conduct, and reporting results and is classified as low, moderate, serious, and critical risk ( 22 ).

### Data Analyses

All analyses were performed using MedCalc for Windows, version 19.4 (MedCalc Software, Ostend, Belgium). The primary outcome was extracted from all included studies. Secondary outcomes were not available in all studies. We calculated each study’s odds ratio (OR) and 95% confidence interval (CI) to evaluate their differences. Chi-squared test and I2 were used to assess heterogeneity. When heterogeneity was present, the random effects model was used. The alpha risk was defined as < 0.05.

## RESULTS

### Search results and selection process

**Figure 1:**
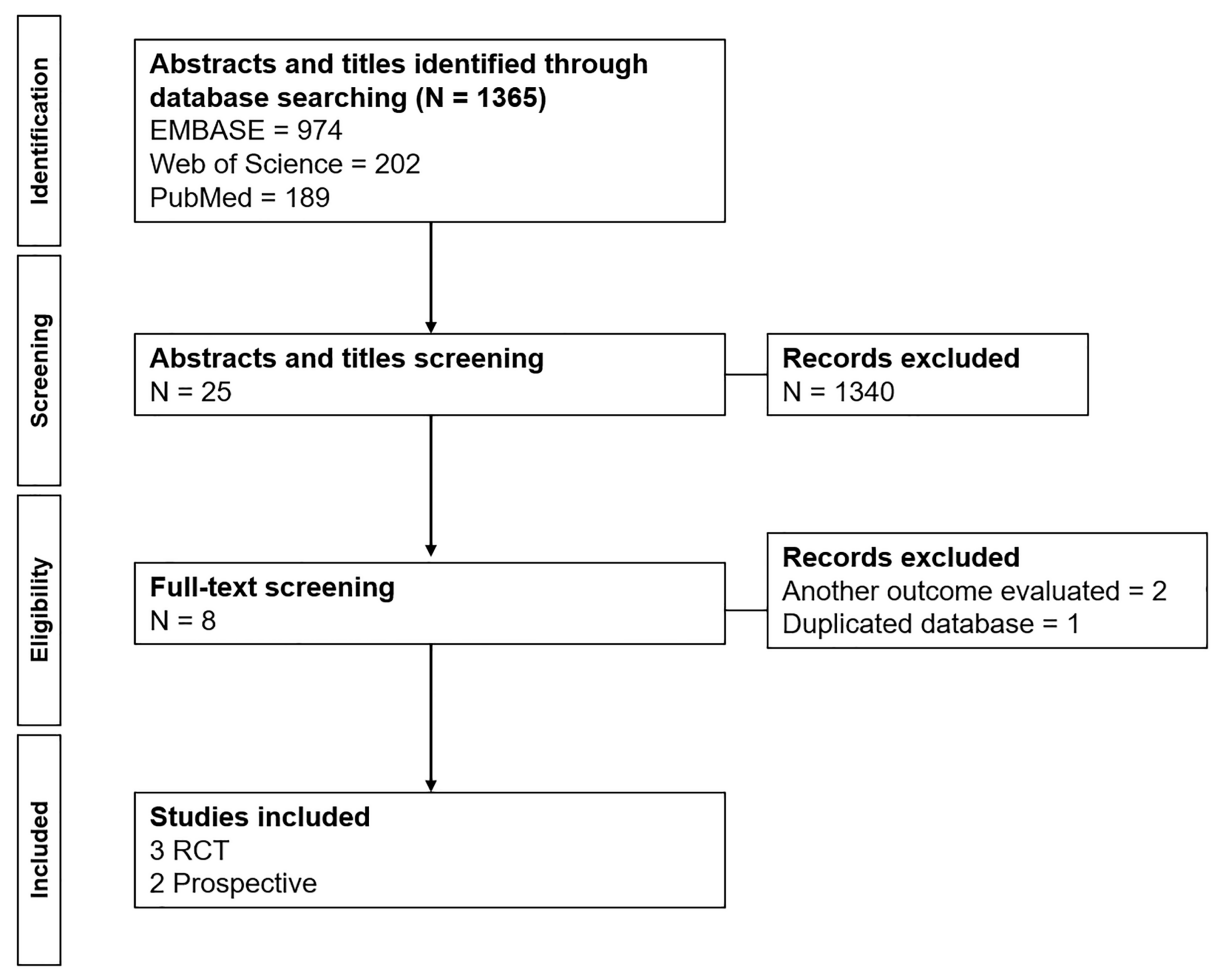
– PRISMA flowchart.

As shown in Figure-1 , literature search identified 1362 publications. Abstracts and titles were screened, excluding all studies that were not prospective or RCT. After full-text screening, eight articles were selected, and three were excluded (another outcome evaluated, and duplicated database). The final selection included five articles (three RCT and two prospective studies) with a total of 475 patients studied.

### Risk of bias

**Figure 2:**
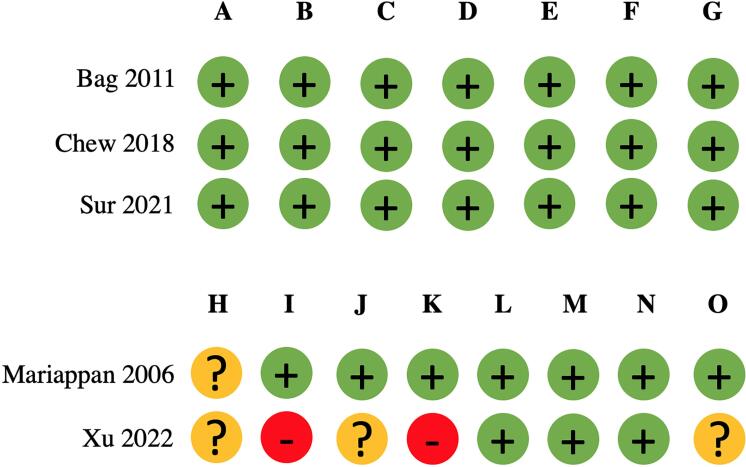
Risk of bias of randomized controlled trials.

As shown in Figure-2 , Bag 2011, Chew 2018, and Sur 2021 were considered to have a low risk of bias in all criteria according to RoB 2 ( 16 , 17 , 23 ). Mariappan 2006 and Xu 2022 were considered to have some moderate/serious risk of bias according to ROBINS-I ( 15 , 24 ). Xu 2022 did not have specific criteria for antimicrobial choice – “antibiotics (type and duration) were given at the discretion of the surgeon; the urine culture took 48-72h, and some patients did not get the results before the procedure” ( 24 ).

(A) Random sequence generation (selection bias); (B) Allocation concealment (selection bias); (C) Blinding of participants and personnel (performance bias); (D) Blinding of outcome assessment (detection bias); (E) Incomplete outcome data (attrition bias); (F) Selective reporting (reporting bias); (G) Other bias; (H) Bias due to confounding; (I) Bias in selection of participants into the study; (J) Bias in classification of interventions; (K) Bias due to deviations from intended interventions; (L) Bias due to missing data; (M) Bias in measurement of outcomes; (N) Bias in selection of the reported result; (O) Overall bias.

### Characteristics of included studies

Mariappan et al. 2006 were the first to demonstrate in a prospective study that one week of antibiotics in patients with high infectious risk undergoing PCNL reduces urosepsis. Results showed a three times less chance of urosepsis in patients receiving antibiotics one week before intervention (RR 2.9; 95% CI 1.3-6.3, p = 0.004)( 15 ).

Bag et al. demonstrated in a RCT of 110 patients with stones ≥ 25 mm or hydronephrosis undergoing PCNL that prophylaxis with nitrofurantoin 100 mg twice daily for a week before PCNL prevents urosepsis and fever. Results showed that patients using nitrofurantoin had less SIRS (19% vs. 49%, OR 0.31, p = 0.01), less positive pelvic urine culture (0 vs. 9.8%, RR 4.95, p = 0.001), and less positive stone culture (8.3% vs. 30.2%, OR 0.22, p = 0.016) ( 16 ).

The EDGE Consortium reported two multicenter RCTs addressing preoperative oral antibiotics in patients undergoing PCNL. Chew et al. conducted a RCT with patients with sterile preoperative urine cultures and no urinary drains, which was deemed “low risk.” There was no difference in the incidence of sepsis (12 vs. 14%, p = 1.0), fever (0 vs. 2.3%, p = 0.24), positive intraoperative renal pelvis urine culture (9.3 vs. 9.3%, p = 1.0) and positive stone culture (2.3 vs. 2.3%, p = 1.0) between antibiotic and control groups ( 23 ). In the EDGE Consortium’s subsequent publication, Sur et al. demonstrated that seven days vs. two days of preoperative 100 mg nitrofurantoin twice daily decreases the risk of urosepsis in moderate to high infectious risk patients undergoing PCNL. Both groups received intravenous antibiotics at the induction of the procedure. It was observed that patients who received two days of antibiotics had a higher risk of sepsis (OR 3.1, 95% CI 1.1 - 8.9, p = 0.031) ( 17 ).

Xu et al. 2022 ( 24 ), prospectively studied the optimal duration of preoperative antibiotic therapy was prospectively studied in consecutive patients with positive urine culture submitted to PCNL. In this “real-world” study, authors concluded that ≥ 7 days of antibiotics before procedure in high infectious risk patients reduces the risk for urosepsis. A significant limitation of this study is that a wide range of antibiotics was used according to sensitivity test of positive urine culture of patients undergoing the procedure. We managed to extract data from patients that used single-dose (28 patients) vs. seven-day (30 patients) antibiotics before PCNL to include in our meta-analysis. It was evidenced that receiving antibiotics seven or more days before the procedure was a protective factor independently associated with SIRS ( 24 ) ( Table-1 ).

**Table 1:** Description of included studies.

Study	Country	Design	Inclusion criteria	Definition of SIRS or Sepsis	Procedure	Patients, n	Mean age, years	Stone size, mm
Mariappan, et al. 2006 ( 15 )	UK	Prospective	Stones ≥ 20 mm and/or dilated pelvicalyceal system	SIRS was defined as the systemic response to infection, manifested by two or more of the following conditions as a result of infection: - Temperature ≥ 38^o^ C or ≤ 36^o^ C - Heart rate > 100 beats/min - Respiratory rate > 20/min - White blood cell count > 12,000 white blood cells/mL or < 4,000 white blood cells/mL	7 days of antibiotic (250 mg of ciprofloxacin twice daily) before PCNL vs. No antibiotic before PCNL	52 vs. 46	55.5 vs. 53.1	30.8 vs. 32.8
Bag et al. 2011 ( 16 )	India	RCT	Patients with stones ≥ 2,5 cm and/or hydronephrosis and sterile urine	Hard criteria for SIRS were fever > 38^o^ C and/or leukocyte counts > 12,000	7 days of antibiotic (100 mg of nitrofurantoin twice daily) before PCNL vs. No antibiotic before PCNL	48 vs. 53	39.2 vs. 40.4	34.1 vs. 36.7
Chew et al. 2018 ( 23 )	USA/ Canada	RCT	Patients with sterile urine and no urinary drain	Sepsis was defined as having an infection source in addition to 2 or more of the following criteria at least 12 hours after the procedure: temperature > 38,3^o^C or < 36^o^C, heart rate > 90/minute, respiratory rate > 20/minute, altered mental status, systolic blood pressure < 90mmHg, mean arterial pressure decrease of more than 40 mmHg and white blood cell count > 12,000 or < 4,000.	7 days of antibiotic (100 mg of nitrofurantoin twice daily) before PCNL vs. No antibiotic before PCNL	43 vs. 43	56 vs. 62	19 vs. 17
Sur et al. 2021 ( 17 )	USA/ Canada	RCT	Patients ≥ 18 years old who had stone burden of any size for which PCNL was recommended. Subjects had to have had either a positive preoperative urine culture within 3 months of the planned procedure or an internalized ureteral stent, nephrostomy tube or nephroureteral stent at time of PCNL.	Sepsis was defined as having an infection source in addition to 2 or more of the following criteria at least 12 hours after the procedure: temperature > 38,3^o^C or < 36^o^C, heart rate > 90/minute, respiratory rate > 20/minute, altered mental status, systolic blood pressure < 90mmHg, mean arterial pressure decrease of more than 40 mmHg and white blood cell count > 12,000 or < 4,000.	7 days of antibiotic (100 mg of nitrofurantoin twice daily) before PCNL vs. 2 days of antibiotic (100 mg of nitrofurantoin twice daily) before PCNL	68 vs. 55	61 vs. 54	20 vs. 23
Xu et al. 2022 ( 24 )	China	Prospective	Patients with positive urine culture undergoing primary PCNL	SIRS was defined as the co-existence of at least two of the following items during the whole hospitalization: temperature > 38^o^C or < 36^o^C, heart rate > 90/min, respiratory rate > 20/min or PaCO2 < 32 mmHg, and white blood cell count > 12,000 or < 4,000.	7 days of empiric antibiotic before PCNL vs. No antibiotic before PCNL	30 vs. 28	NA	NA

SIRS = systemic inflammatory response syndrome; PCNL = Percutaneous Nephrolithotomy; RCT = randomized controlled trials; NA = not available

### Outcomes

**Figure 3:**
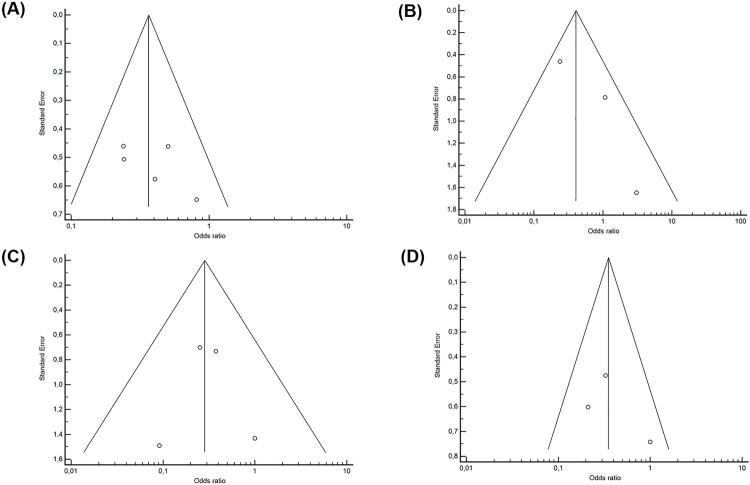
– Funnel plot – (A) patients with SIRS or sepsis; (B) patients with fever; (C) positive intraoperative urine; (D) positive stone culture.

**Figure 4:**
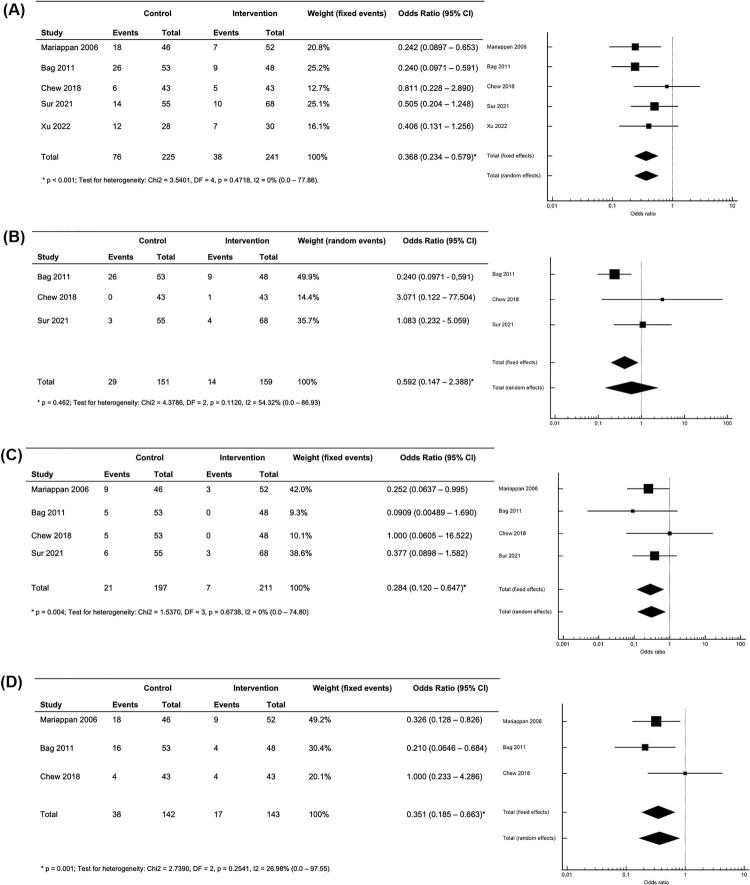
– Forest plot – (A) SIRS or sepsis in control vs. intervention; (B) fever in control vs. intervention; (C) positive intraoperative urine culture in control vs. intervention; (D) positive stone culture in control vs. intervention.

SIRS/sepsis outcome was retrieved from all studies included. Postoperative fever outcome was extracted from three studies. Intraoperative urine culture and stone culture outcomes were extracted from four and three studies, respectively. Funnel plots demonstrating studies’ bias and heterogeneity are shown in Figure-3 . Forest plots ( Figure-4 ) evidenced that using antibiotics for seven days in the preoperative period of PCNL was a protective factor for developing SIRS/sepsis (OR 0.366, 95% CI 0.234 - 0.527, p < 0.001). There was no statistical association between the seven-day use of antibiotics and fever (OR 0.592, 95% CI 0.147 – 2.388, p = 0.462). Patients who received the intervention had lower positive intraoperative urine culture (OR 0.284, 95% CI 0.120 – 0.674, p = 0.004) and stone culture (OR 0.351, 95% CI 0.185 – 0.663, p = 0.001) than the control group.

## DISCUSSION

This meta-analysis shows that seven days of oral preoperative antibiotics plus a dose of intravenous antibiotics at the time of surgery reduces the risk of infection in patients undergoing PCNL. Extended preoperative antibiotic use reduced the risk of SIRS and positive intraoperative urine culture and stone culture, regardless of the patient’s risk of infection. Due to a lack of consensus in defining high infectious risk patients for PCNL, this meta-analysis included all adult patients undergoing PCNL. Our meta-analysis included only studies that investigated preoperative and not postoperative use of antibiotics to avoid confounding timing in antibiotics use in patients undergoing PCNL. The previous meta-analysis joined studies of preoperative and postoperative use of antibiotics, reducing its clinical application ( 8 ).

Nowadays, sepsis definition is as a life-threatening organ dysfunction caused by a dysregulated host response to infection ( 25 ). However, in the past, sepsis was described as a systemic inflammatory response syndrome (SIRS) to infection ( 19 ). In some studies, researchers referred to urosepsis as SIRS resulting from infection in the urinary tract in patients undergoing PCNL. Mariappan et al. and Bag et al. considered SIRS as fever > 38º C and/or leukocyte counts > 12,000 and attributed to urosepsis after excluding perinephric collection, pleural effusion, chest infection, and thrombophlebitis ( 15 , 16 ). The EDGE Consortium used the more current definition of sepsis, which includes two or more of the following criteria at least 12 hours after the procedure: temperature above 38.3ºC or below 36ºC, heart rate above 90/minute, respiratory rate greater than 20/minute, altered mental status, systolic blood pressure less than 90 mmHg, mean arterial pressure less than 70 mmHg or systolic blood pressure decrease of more than 40 mmHg, and white blood cells greater than 12,000 or less than 4,000 ( 17 , 23 ). Despite the definition used at the time of performance of the study, researchers investigated whether preoperative antibiotics could prevent infection, and the incidence of this event was similar between studies. This was the main reason we maintained the definition of sepsis in each original study.

We choose to include in this meta-analysis adult patients undergoing PCNL regardless of their risk of infection. The definition of high infectious risk patients for PCNL varies among studies and is controversial. Patients with sterile urine and dilated pelvicalyceal systems and/or stones of ≥ 20 mm were considered at high infectious risk by Mariappan et al. based on a previous publication from their group ( 26 ). Other authors considered sterile urine, hydronephrosis, and/or stones ≥ 25 mm high risk ( 16 ). However, it is unclear if those patients had positive urine culture weeks before PCNL and were treated. In contrast to Mariappan et al. and Bag et al., stone size or dilated collecting system were not considered risk factors in the Sur et al. study. A previous RCT of the EDGE group did not demonstrate a benefit for the preoperative use of nitrofurantoin for seven days in patients with sterile urine and no urinary drain undergoing PCNL ( 23 ). Therefore, EDGE Consortium created a definition of moderate to high infectious risk patients with a positive preoperative urine culture within three months of the planned procedure or an internalized ureteral stent, nephrostomy tube, or nephro-ureteral stent at the time of surgery ( 17 ). Xu et al. considered patients receiving antibiotic treatment for a positive urine culture, regardless of stone size, as high infectious risk patients for PCNL ( 24 ).

It was consensual amongst investigators that the choice of which antibiotic to use preoperatively in patients undergoing PCNL should be based on local bacterial sensitivity patterns ( 15 - 17 , 23 , 24 ). Mariappan et al. chose ciprofloxacin, while Bag et al., Chew B et al., and Sur et al. chose nitrofurantoin ( 15 - 17 , 23 ). Although the level of bacterial resistance to nitrofurantoin is low, it is essential to note that nitrofurantoin has poor penetration into the tissues, and *Proteus sp* . and *Pseudomonas sp* . have inherited chromosomal resistance to it ( 27 - 29 ).

This meta-analysis demonstrated the protective role of one week of preoperative oral antibiotics for patients undergoing PCNL. Still, we recognize limitations, including a low number of subjects, heterogeneity of definitions of sepsis, and antibiotic use. The low number of participants is explained by our strict inclusion criteria of only prospective or randomized controlled trials in this meta-analysis. Nevertheless, the quality of a meta-analysis depends on the quality of the original studies included. As we aimed to investigate whether an intervention could reduce the risk of a serious complication, it was essential to have only prospective data due to its reliability and to minimize selection and report bias ( 30 ). Retrospective studies tend to underreport complications compared to their prospective counterparts. The definition of sepsis is an ongoing process, and we choose to keep the author´s definition at the time of the performance of the study. It is impossible to define the best prophylactic antibiotic based on this meta-analysis. Although the antibiotic used varied among studies, authors preferred ciprofloxacin or nitrofurantoin based on local bacterial flora.

## CONCLUSIONS

We conclude that one week of prophylactic oral antibiotics based on local bacterial sensitivity pattern plus a dose of intravenous antibiotics at the time of surgery in patients undergoing PCNL reduces the risk of infection. To optimize preoperative antibiotic use, more prospective data are needed to define better which patients are at a higher risk of infection after PCNL.
